# The use of animals in neuroscience research

**DOI:** 10.1093/brain/awag071

**Published:** 2026-02-20

**Authors:** Yvonne Couch

**Affiliations:** Nuffield Department of Clinical Neurosciences, University of Oxford, Oxford OX1 3QU, UK

## Abstract

Amid growing international efforts to reduce animal use in research, Couch explores the implications for neuroscience. She argues that moving away from *in vivo* models poses major scientific challenges, as whole-organism insights into brain circuitry and brain-body integration are difficult to replicate *in vitro*.

***Reducing animal research in neuroscience risks losing whole-organism insight that is challenging to capture* in vitro.**

In November 2025, the UK Government unveiled a strategy to accelerate the transition away from animal testing, aiming to reduce animal use and invest in human-relevant research methods. For neuroscience, a discipline which has traditionally been dependent on *in vivo* models to understand the brain’s intricate circuitry and its integration with the body, this initiative will (whilst being ethically commendable) provide a major scientific challenge. As a strategy, it presents a compelling vision for 21st-century biomedical research, but it requires researchers to engage proactively to ensure that a reduction in animal use does not inadvertently compromise the rigour or translational relevance of *in vivo* neuroscience.

**Figure:**
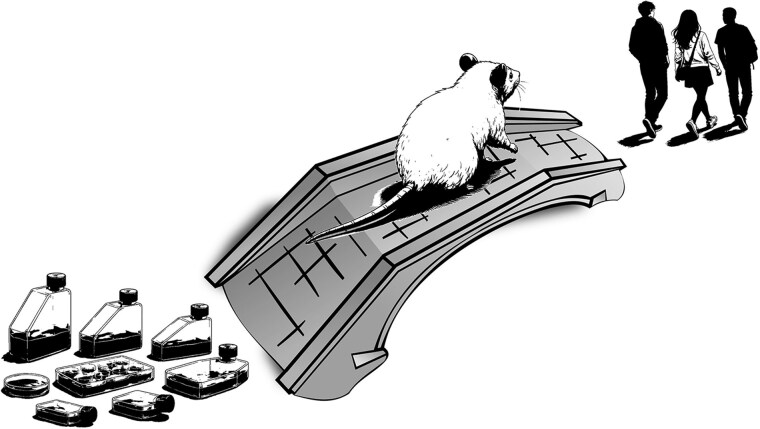


Animal use in scientific research in the UK is held to an extremely high standard. ASPA, or the Animals (Scientific Procedures) Act was introduced in 1986 and regulates the use of animals in research at the institutional, group and personal level. A 2002 study by the House of Lords stated that the UK held its animal researchers to higher standards than any other country in the world.^1^ UK institutions are extremely transparent about their animal use, with numbers of animals used in scientific procedures being readily available to those wishing to research the details.^2^ This is not the case for many other countries, where animal numbers are simply not reported or not reported on an institutional level.^1,2^

Animal use in research in the UK is also falling. According to recent reports, use in 2024 was 77% of what it was in 2019.^2^ Around 1.4 million animals were used in procedures, and 1.2 million were involved in the generation and breeding of genetically altered lines. For context, estimates show that around 45 million male chicks are killed each year in the UK because they are of no use for breeding or meat production,^3^ and domesticated cats are estimated to injure or kill over 100 million animals per year.^4^ And we learn very little from those animals. In contrast, there are countless examples of excellent translational pathways from rodent models to the clinic.

In stroke, for example, models of focal ischaemia were instrumental in establishing thrombolysis as a viable therapeutic concept.^5-7^ Studies in rats and mice using recombinant tissue plasminogen activator demonstrated reduced infarct volumes and improved outcomes when it was given rapidly after occlusion.^8,9^ These data underpinned the NINDS clinical trial that led to the approval of intravenous alteplase for acute ischaemic stroke, one of the clearest examples of preclinical neuroscience translating directly to a standard-of-care therapy.^10^ But it should be noted that not all animal research translates well. According to reports, over 1000 preclinical studies in stroke have resulted in around 100 clinical trials, none of which have translated into therapy (see the Supplementary material for additional reading).

However, this poor translation of preclinical findings should not be taken as evidence that animal models are intrinsically uninformative, but rather that experimental design, reporting, and model selection are critical determinants of translational success. Substantial progress has been made in improving the rigour of *in vivo* neuroscience through preregistration, randomization, blinding and the routine consideration of sex, age and comorbidities. The application of such principles, alongside transparent reporting frameworks such as ARRIVE, has already improved many areas of preclinical research. The challenge now is to integrate this improved version of traditional animal research with emerging *in silico*, *in vitro* and microphysiological models, leveraging the strengths of each while recognizing and mitigating their respective limitations.

Each of these complementary systems offers unique opportunities to strengthen the translational pipeline. *In silico* approaches allow us to model pharmacokinetics, receptor dynamics and molecular interactions at a scale and speed that are impossible *in vivo*, guiding compound design before a single dose is administered to anything or anyone. In practice, *in silico* methods are already central to modern translational pipelines. Virtual screening, docking and machine-learning-based scoring reduce the number of compounds advanced to *in vitro* and *in vivo* testing; PBPK/PD (physiologically based pharmacokinetic/pharmacodynamic) models inform first-in-human dosing and drug-drug interaction assessment and are now accepted by regulators in defined contexts. Breakthroughs in protein modelling (AlphaFold) plus generative AI have further accelerated target engagement and molecule ideation. *In silico* predictions are an excellent start to the preclinical pipeline and are used to efficiently triage and refine candidates.

Once a candidate has been developed *in silico*, basic testing can be carried out *in vitro*. *In vitro* systems, ranging from classical cell lines to patient-derived induced pluripotent stem cell-derived cultures, provide an ethically sustainable platform for pathway validation. In drug discovery, *in vitro* assays are typically the first experimental step after computational triage, confirming predicted target engagement, estimating potency and identifying early liabilities, such as cytotoxicity or off-target binding. These data form the foundation of quantitative structure–activity relationships and can feed directly back into model refinement *in silico*.

Recent advances in stem-cell biology now allow *in vitro* systems to recapitulate many aspects of human CNS cell physiology that were previously inaccessible. Human induced pluripotent stem cells (hiPSCs) from patients can be differentiated into specific neuronal or glial subtypes, enabling disease-relevant assays for conditions such as amyotrophic lateral sclerosis, Parkinson’s disease and Alzheimer’s disease. These cultures have already demonstrated predictive value for drug response and toxicity, and papers have identified some pharmacological sensitivities that were not evident in rodent neurons. However, whilst iPSC-based models offer human-cell contexts and bypass fundamental species differences, they are not without their own pitfalls. Many studies rely on a small number of iPSC lines, often omit donor sex or donor background reporting, and use sample sizes that are too small to robustly account for inter-line variability. These lines are, essentially, individual humans in a dish and we would be reluctant to endorse studies in humans with such low *n* numbers and no patient information. These limitations must be recognized if iPSC systems are to fulfil their potential as rigorous translational tools, rather than being over-interpreted without appropriate validation or integration with clinical and *in vivo* data.

*In vitro* studies have traditionally been inexpensive and scalable, although this is less true with the advent of iPSC cultures. They also do not fully capture the complexity of the human organ. The brain, in particular, has more different types of cells in it than any other organ in the body. Organoids and organ-on-chip technologies offer to bridge the gap between reductionist assays and the complexity of whole organisms.

Organ-on-chip technologies represent an evolution of the traditional *in vitro* approach, designed to reproduce key aspects of tissue microenvironment and organ-level physiology under controlled conditions. These microfluidic systems incorporate human cells (often iPSCs and disease-relevant models) within precisely engineered channels that permit continuous perfusion, mechanical stimulation and the establishment of physiological gradients. As a result, they can model dynamic functions such as neurite outgrowth, neurovascular coupling, barrier permeability, vascular shear stress or neuromuscular signalling, which are largely absent from static cultures.

The translational value of organ-on-chip systems lies in their ability to generate human-relevant data at the interface of biology and engineering. For example, blood–brain barrier chips have been used to investigate transport kinetics and permeability of CNS drugs, correlating closely with *in vivo* human data. Multi-organ or ‘body-on-chip’ platforms now allow sequential coupling of liver, gut, kidney and brain modules, recreating drug metabolism and systemic distribution without the need for animal models. Indeed, regulatory agencies are beginning to recognize their potential with both the US FDA (Federal Drug Administration) and EMA (European Medicines Agency) developing guidelines and working groups for microphysiological systems, and liver chips in particular are beginning to be used as a possible screening tool for basic toxicity studies.

Despite this, organ-on-chip systems remain constrained by practical and biological limitations. The cells incorporated into these platforms are often specially selected for their ability to survive under identical culture conditions, rather than for their physiological fidelity. As such, true multi-organ integration requires a compromise between cell-type diversity and media compatibility, which can distort metabolic or signalling pathways. Fluidic coupling between modules can reproduce circulation, but it rarely captures immune surveillance (immune cells moving in and out of tissues), hormonal feedback, or the stochastic variability and inherent noise present in whole organisms. Engineering constraints (use of polydimethylsiloxane) and limited scalability further restrict their current use. Finally, reproducibility remains a challenge (as it does in many areas of science) where variations in chip design, flow rates, or cell sourcing can yield significant inter-lab variability in data.

Whereas organ-on-chip systems attempt to recreate physiology through external engineering, organoids approach the same goal from within, relying on the intrinsic self-organization of human cells into 3D, tissue-like structures. Derived from pluripotent stem cells or organ-restricted progenitors, organoids recapitulate many aspects of human development, cellular diversity and spatial organization that are absent from 2D cultures.^3^ Brain organoids, for example, can generate layered cortical architecture, spontaneous electrophysiological activity and region-specific neuronal subtypes, providing a window into human neurodevelopment. And the translational appeal is obvious; organoids are genetically human, can incorporate patient-specific iPSC lines, and allow the study of disease mechanisms or therapeutic responses in a personalized context.

Yet organoids, too, have significant constraints. Many lack vasculature, immune or endocrine components often leaving organoids with, essentially, an ischaemic core. Reproducibility is hampered by the absence of standardized differentiation protocols and by selective reporting of successful cultures. Moreover, the long culture times, cost, and expertise required currently preclude high-throughput use. As with organ-on-chip platforms, the key will be integration, using organoids to explore human-specific mechanisms and validate *in vitro* and *in silico* predictions whilst being aware of their limitations.

But even the most sophisticated *in vitro* or *in silico* systems cannot currently recapitulate the integrated physiology of a living organism. The University of Oxford’s policy on the use of animals in scientific research states that such work should be carried out only when no other alternative is available. This principle reflects scientific pragmatism rather than a moral loophole, certain questions simply cannot currently be answered in any other way. My own field (brain–immune communication) is a case in point. The bidirectional exchange between the CNS and the immune system involves dynamic signalling across the blood–brain barrier, neurovascular and lymphatic interfaces, hormonal cascades and behavioural feedback. Capturing these interactions requires both a functioning brain and immune system. Beyond this basic requirement, much of the work I undertake is to understand mechanisms. This requires a degree of experimental control (time points, comorbidities, age, sex, etc.) which is simply not ethically feasible in a patient population. By combining my animal studies with clinical samples and non-animal alternatives, I aim to use animals in context rather than in isolation, and only when no other alternative exists, as the indispensable bridge between molecular insight and whole-organism function.

But beyond my own field, the same holds true for other areas of neuroscience research and other neuroscience researchers. Circadian biology, for instance, depends on the orchestration of neural, endocrine and metabolic rhythms across multiple organs; the study of diet and gut–brain interactions involve nutrient flux, microbial metabolism and systemic immune responses. These phenomena emerge from whole-body integration and are, at present, beyond the reach of even the most advanced organoids or microphysiological chips. For such questions, ethically conducted animal research remains essential. Not necessarily as a default, but as a carefully justified component of a broader translational research ecosystem in which computational, cellular and animal approaches inform one another. Reducing animal work indiscriminately, without regard for what questions can and cannot yet be answered by alternative models, would risk slowing both scientific and clinical progress.

Looking forward, the most productive path for neuroscience is unlikely to involve replacing animal models with any single alternative technology, but rather integrating computational, cellular, and *in vivo* approaches in a complementary and hypothesis-driven manner. *In silico* and *in vitro* systems can be used to refine questions, reduce exploratory animal use and prioritize the most informative experiments, while *in vivo* studies remain essential for testing mechanisms that depend on whole-organism physiology. Such an integrated framework aligns both with ethical imperatives to reduce animal use and with the scientific need to preserve translational relevance.

## Supplementary Material

awag071_Supplementary_Data

## Notes

Yvonne Couch is an Associate Professor of Neuroimmunology at the University of Oxford, studying how inflammation and vascular pathology shape long-term brain health after injuries such as stroke and in dementia.
