# Supplementary material for: The use of animals in neuroscience research
Source: Brain. 2026 Feb 20;149(7):2197–9. doi: 10.1093/brain/awag071 (PMC13337216; doi:10.1093/brain/awag071)
Supplement: awag071_Supplementary_Data [file awag071_supplementary_data.pdf]

## Additional reading


Neuhaus, A. A. *et al.* Importance of preclinical research in the development of neuroprotective strategies for ischemic stroke. *JAMA Neurol* **71**, 634-639, doi:10.1001/jamaneurol.2013.6299 (2014).

Neuhaus, A. A., Couch, Y., Hadley, G. & Buchan, A. M. Neuroprotection in stroke: the importance of collaboration and reproducibility. *Brain* **140**, 2079-2092, doi:10.1093/brain/awx126 (2017).

Schmidt-Pogoda, A. *et al.* Why Most Acute Stroke Studies Are Positive in Animals but Not in Patients: A Systematic Comparison of Preclinical, Early Phase, and Phase 3 Clinical Trials of Neuroprotective Agents. *Ann Neurol* **87**, 40-51, doi:10.1002/ana.25643 (2020).

Kilkenny, C., Browne, W., Cuthill, I. C., Emerson, M. & Altman, D. G. Animal research: reporting in vivo experiments: the ARRIVE guidelines. *J Gene Med* **12**, 561-563, doi:10.1002/jgm.1473 (2010).

Guidance, F. The use of physiologically based pharmacokinetic analyses—biopharmaceutics applications for oral drug product development, manufacturing changes, and controls. *Center for Drug Evaluation and Research (CDER)* (2020).

Leedale, J. *et al.* A Combined In Vitro/In Silico Approach to Identifying Off-Target Receptor Toxicity. *iScience* **4**, 84-96, doi:10.1016/j.isci.2018.05.012 (2018).

Valadez-Barba, V. *et al.* iPSC for modeling neurodegenerative disorders. *Regen Ther* **15**, 332-339, doi:10.1016/j.reth.2020.11.006 (2020).

Druwe, I., Freudenrich, T. M., Wallace, K., Shafer, T. J. & Mundy, W. R. Comparison of human induced pluripotent stem cell-derived neurons and rat primary cortical neurons as in vitro models of neurite outgrowth. *Applied in vitro Toxicology* **2**, 26-36 (2016).

Moakley, D. *et al.* Pharmacological Profiling of Purified Human Stem Cell-Derived and Primary Mouse Motor Neurons. *Sci Rep* **9**, 10835, doi:10.1038/s41598-019-47203-7 (2019).

Herculano-Houzel, S. The human brain in numbers: a linearly scaled-up primate brain. *Front Hum Neurosci* **3**, 31, doi:10.3389/neuro.09.031.2009 (2009).

Lyck, R., Nishihara, H., Aydin, S., Soldati, S. & Engelhardt, B. Modeling Brain Vasculature Immune Interactions In Vitro. *Cold Spring Harb Perspect Med* **13**, doi:10.1101/cshperspect.a041185 (2023).

Kantawala, B. *et al.* Revolutionizing neurotherapeutics: blood-brain barrier-on-a-chip technologies for precise drug delivery. *Ann Med Surg (Lond)* **86**, 2794-2804, doi:10.1097/MS9.0000000000001887 (2024).

Herland, A. *et al.* Quantitative prediction of human pharmacokinetic responses to drugs via fluidically coupled vascularized organ chips. *Nat Biomed Eng* **4**, 421-436, doi:10.1038/s41551-019-0498-9 (2020).

Shroff, T. *et al.* Studying metabolism with multi-organ chips: new tools for disease modelling, pharmacokinetics and pharmacodynamics. *Open Biol* **12**, 210333, doi:10.1098/rsob.210333 (2022).

Administration, U. S. F. a. D. Roadmap to Reducing Animal Testing in Preclinical Safety Studies. (2023).

26 Agency, E. M. First EMA workshop on non-animal approaches in support of medicinal product development – challenges and opportunities for use of micro-physiological systems. (2018).

Jang, K. J. *et al.* Reproducing human and cross-species drug toxicities using a Liver-Chip. *Sci Transl Med* **11**, doi:10.1126/scitranslmed.aax5516 (2019).

Ewart, L. *et al.* Performance assessment and economic analysis of a human Liver-Chip for predictive toxicology. *Commun Med (Lond)* **2**, 154, doi:10.1038/s43856-022-00209-1 (2022).

Picollet-D'hahan, N., Zuchowska, A., Lemeunier, I. & Le Gac, S. Multiorgan-on-a-Chip: A Systemic Approach To Model and Decipher Inter-Organ Communication. *Trends Biotechnol* **39**, 788-810, doi:10.1016/j.tibtech.2020.11.014 (2021).

Low, L. A. & Tagle, D. A. Organs-on-chips: Progress, challenges, and future directions. *Exp Biol Med (Maywood)* **242**, 1573-1578, doi:10.1177/1535370217700523 (2017).

Shirure, V. S. & George, S. C. Design considerations to minimize the impact of drug absorption in polymer-based organ-on-a-chip platforms. *Lab Chip* **17**, 681-690, doi:10.1039/c6lc01401a (2017).

Grant, J. *et al.* Simulating drug concentrations in PDMS microfluidic organ chips. *Lab Chip* **21**, 3509-3519, doi:10.1039/d1lc00348h (2021).

Nie, X. *et al.* Novel organoid model in drug screening: Past, present, and future. *Liver Res* **5**, 72-78, doi:10.1016/j.livres.2021.05.003 (2021).

Lancaster, M. A. *et al.* Cerebral organoids model human brain development and microcephaly. *Nature* **501**, 373-379, doi:10.1038/nature12517 (2013).

Quadrato, G. *et al.* Cell diversity and network dynamics in photosensitive human brain organoids. *Nature* **545**, 48-53, doi:10.1038/nature22047 (2017).

Andrews, M. G. & Kriegstein, A. R. Challenges of Organoid Research. *Annu Rev Neurosci* **45**, 23-39, doi:10.1146/annurev-neuro-111020-090812 (2022).

Ahammed, B. & Kalangi, S. K. A Decade of Organoid Research: Progress and Challenges in the Field of Organoid Technology. *ACS Omega* **9**, 30087-30096, doi:10.1021/acsomega.4c03683 (2024).
